# Kinetics of *BCR::ABL1* transcript levels and molecular relapse after tyrosine kinase inhibitors discontinuation in chronic myeloid leukemia patients: preliminary results from the DES-CML study

**DOI:** 10.3389/fonc.2024.1393191

**Published:** 2024-05-08

**Authors:** Bruna Murbach, Gislaine Duarte, Leonardo Carvalho Palma, Eliana Miranda, Guilherme Duffles, Graziele Pavan Furlin, Isabella Toni, Carmino De Souza, Larissa Binelli, Vitor Leonardo Bassan, Fabiola Attie de Castro, Lorena Lobo de Figueiredo-Pontes, Katia Borgia Barbosa Pagnano

**Affiliations:** ^1^ Centro de Hematologia e Hemoterapia (Hemocentro-UNICAMP), Universidade Estadual de Campinas (UNICAMP), Campinas, São Paulo, Brazil; ^2^ Hematology Division, Department of Medical Images, Hematology, and Clinical Oncology, Ribeirão Preto Medical School, University of São Paulo, Ribeirão Preto, Brazil; ^3^ Department of Clinical Analyses, Toxicology and Food Science, School of Pharmaceutical Sciences of Ribeirão Preto, University of São Paulo, Ribeirão Preto, Brazil

**Keywords:** Chronic myeloid leukemia, tyrosine kinase inhibitors, discontinuation, dose de-escalation, BCR::ABL1 transcripts, molecular relapse

## Abstract

Tyrosine kinase inhibitors (TKI) have revolutionized the treatment of patients with chronic myeloid leukemia. Patients who achieve sustained deep molecular response are eligible for treatment discontinuation. DES-CML is an ongoing, phase 2 multicentric discontinuation trial. Adult patients with CML in chronic phase with typical *BCR::ABL1* transcripts, stable deep molecular response (MR4.5 IS) for two years, and no previous resistance were eligible. Patients underwent a phase of TKI dose de-escalation for six months before discontinuation. TKI was reintroduced at the previous dose if the patient lost major molecular response (MMR) at any time. This study aimed to assess the impact of BCR-ABL transcript kinetics during TKI de-escalation and discontinuation phases on treatment-free survival. So far, the study recruited 41 patients, and 38 patients discontinued therapy (4 were in the second discontinuation attempt). Eleven patients lost MMR, one during the de-escalation phase and ten after discontinuation. 24-month treatment-free survival was 66% (95% CI: 48-84%) in a median follow-up of 7 (1–30) months. No patient lost hematological response or had disease progression. A higher rate of molecular relapses occurred in patients with fluctuating *BCR::ABL1* levels after the discontinuation phase (with loss of MR4.5, but no loss of MMR) (P=0.04, HR-4.86 (1.03-22.9) but not confirmed in the multivariate analysis. The longer duration of TKI treatment (P=0.03, HR-1.02, 95%CI - 1.00-1.04) and MMR (P=0.004, HR-0.95, 95%CI - 0.92-098) were independent factors of a lower relapse rate.

## Introduction

Chronic myeloid leukemia (CML) is a myeloproliferative neoplasm characterized by the reciprocal chromosomal translocation t(9;22), which results in the formation of the Philadelphia (Ph) chromosome containing the *BCR::ABL1* gene. This gene encodes a protein with tyrosine kinase activity, a therapeutic target of tyrosine kinase inhibitors (TKI) (1, 2). CML is a disease with an incidence of 1–2 cases per 100,000 adults, accounting for approximately 15% of diagnosed cases of leukemia (3).

The development of TKI changed the natural history of the disease, drastically reducing the rates of transformation to the blastic phase, increasing patient survival from a historical rate of 10-20% to above 80%, resulting in a life expectancy close to that of the normal population, as long as they receive appropriate TKI treatment and maintain treatment adherence (4–7). Several studies confirm that discontinuation of TKI is feasible and safe in patients who achieve a sustained deep molecular response (8, 9). Discontinuation of TKI therapy can be successful in approximately 20% of the total number of CML patients who start therapy (10).

The duration of previous treatment, the depth of molecular response, previous resistance, type of *BCR::ABL1* transcripts, halving time, and lymphocyte subtypes are known predictive factors of molecular recurrence after tyrosine kinase inhibitors (TKI) discontinuation (11–20). Few studies investigated the kinetics of *BCR::ABL1* transcripts during a dose de-escalation phase before discontinuation in predicting molecular relapse (21, 22).

In the present study, we aimed to evaluate the kinetics of *BCR::ABL1* transcripts during the de-escalation and discontinuation phases of the DES-CML TKI discontinuation trial and the impact on treatment-free survival (TFS).

## Methods

The DES-CML study is a multicenter, prospective, open-label, single-arm, phase 2, non-randomized, ongoing trial that enrolled patients with chronic myeloid leukemia treated in the Unified Health System from two Brazilian centers (Study of treatment discontinuation of chronic myeloid leukemia in the Unified Health System - DES-CML Study). This study is registered at the Brazilian Registry of Clinical Trials (ReBEC) platform (RR-6f5xbq; UTN code: U1111-1252-7312), and the ethics committees of the participating centers (Universidade Estadual de Campinas and USP-Ribeirão Preto) approved the protocol. All patients signed informed consent. Inclusion criteria used were adult CML chronic phase patients (18 years or older) receiving first or second-line treatment (if toxicity with the first-line therapy) with TKI for a minimum of 3 years that achieved sustained deep molecular response (DMR), defined by a minimum duration of MR4.5 for two years, confirmed by four real-time quantitative reverse-transcription polymerase chain reaction (qPCR) tests within six months interval. Exclusion criteria were patients with atypical *BCR::ABL1* transcripts, previous or current advanced phase CML, previous resistance to TKI or ABL mutations, and patients with prior allogeneic bone marrow transplantation. The TKI dose was reduced by 50% for six months before discontinuation (de-escalation phase). Molecular monitoring was performed by qPCR, using ABL as the control gene, and the results were reported using the International Scale (23–26). *BCR::ABL1* transcript levels were evaluated monthly during the de-escalation phase. After discontinuation, assessments were performed monthly for six months, every two months at months 7-24, and then every three months. Molecular recurrence definition was the MMR loss (qPCR>0.1%) (11) and was a trigger for treatment reintroduction. TKI was reintroduced at the same dose before dose de-escalation.

### Statistical analysis

The statistical analysis was performed using the IBM-SPSS software. Study data were collected and managed using REDCap (Research Electronic Data Capture) tools hosted at University of Campinas (27). Treatment-free survival (TFS) was calculated from the date of discontinuation until the loss of MMR or TKI reintroduction. The rates of TFS were determined using the Kaplan-Meier method and Log-Rank test for comparison. Gray’s test was also applied to calculate Cumulative Incidence. Death in MMR was considered a competitive event. in significant molecular remission was the only occurring event. The Backward method of Cox regression was used to identify the prognostic factors. In the univariate analysis, a P-value of 0.10 was used to choose the variables for multivariate analysis. P values <.05 were considered statistically significant. The cut-off date for this analysis was November 29, 2023.

## Results

**Figure 1:**
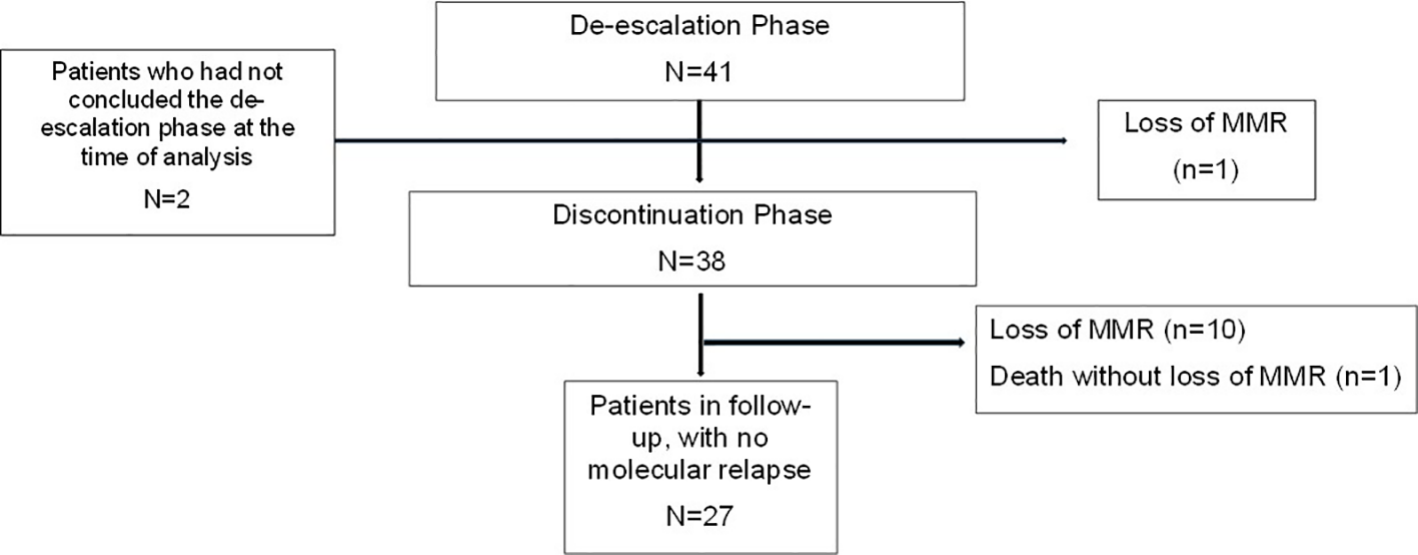
Flow chart of CML patients in the de-escalation and discontinuation phase (n=41).

Between September 2020 and November 2023, 41 CML patients were recruited for the study (**Figure 1**). Four patients were in the second discontinuation attempt, but all were treated with imatinib for at least four years before the second attempt. The patients’ median age at study entry was 62 years (24-79); the majority were male (61%); 35% Sokal low risk, 49% intermediate, 16% high; 79% ELTS low risk, 18% intermediate, 3% high; 82% had b3a2 *BCR::ABL1* transcripts; 86% were using imatinib, 7% nilotinib, 5% dasatinib and 2% bosutinib. The median duration of TKI treatment until the date of discontinuation was 12 years (4-20). CML patient’s characteristics are described in **Table 1**. The median follow-up was 20 months (3-39 months) from the dose de-escalation and seven months (1-30) from the date of discontinuation. Two patients were in the discontinuation phase at the cut-off date (November 2023).

**Table 1:** Clinical and laboratory characteristics of CML patients with chronic myeloid leukemia at diagnosis and study entry (n=41).

Variables	n=41 (%)
Age at diagnosis, years*	50 (9-77)
Age at discontinuation, years* (38 cases)	62 (24-79)
Gender: Male	25 (61)
Sokal score (diagnosis)	
Low	13 (32)
Intermediate	18 (44)
High	6 (14)
Missing	4 (10)
ELTS score (diagnosis)	
Low	30 (73)
Intermediate	7 (17)
High	1 (3)
Missing	3 (7)
*BCR::ABL*1 transcript type	
b3a2	32 (79)
b2a2	6 (15)
b3a2 + b2a2	1 (2)
p210	2 (4)
Previous use of interferon	4 (10)
TKI treatment duration (in years)*	12 (4-20)
Months* between diagnosis and entry into the study	139 (48-299)
MMR Duration (in months)*	143 (46- 216)
MR4.5Duration (in months)*	122 (28-197)

*Median (range).

Forty-one patients de-escalated therapy to half of the previous dose (**Supplementary Table 1**). Two patients did not complete the six months of de-escalation due to lack of medication and went earlier to the discontinuation phase. One patient lost MMR in the de-escalation phase and returned to the previous dose. Two patients were still in the de-escalation phase at the moment of the present analysis. Among the 38 patients that interrupted TKI, 13 (34%) had lost MR4.5 in the de-escalation phase, and 5/13 (38.4%) lost MMR after discontinuation. Among the 25 patients who did not lose MR4.5 during the de-escalation phase, there were five relapses after discontinuation (20.0%).

Ten out of 38 patients lost MMR (26%) after discontinuation. The median time between discontinuation and MMR loss was four months (1-11). The median time for recovering MMR was two months (1-5). All patients recovered their response, and only one did not recover MR4.5.

**Figure 2:**
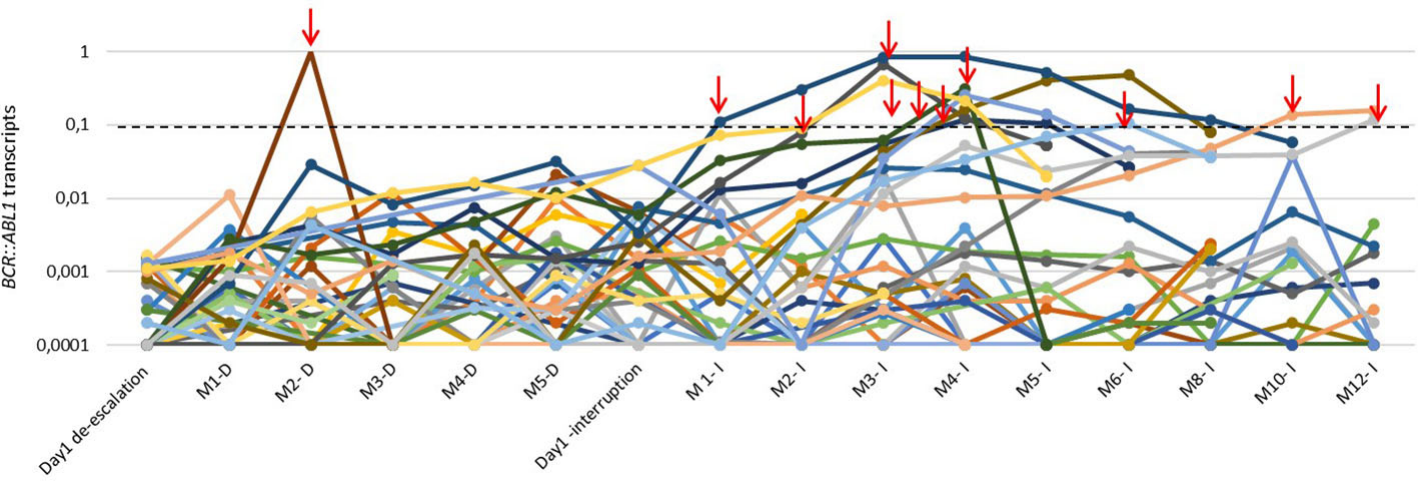
*BCR::ABL1* transcript levels of 41 patients with CML during the de-escalation phase and along the first 12 months of TKI interruption. The red arrows highlight the loss of MMR and treatment reintroduction. M: month; D:de-escalation; I:interruption.

The molecular response status before discontinuation was 30 (79%) RM4.5, 7(18%) RM4.0, and 1 (3%) MMR. There were no relapses after 12 months of treatment interruption. *BCR::ABL1* levels from the first day of TKI dose de-escalation and TKI discontinuation until 12 months are demonstrated in **Figure 2**.

**Figure 3:**
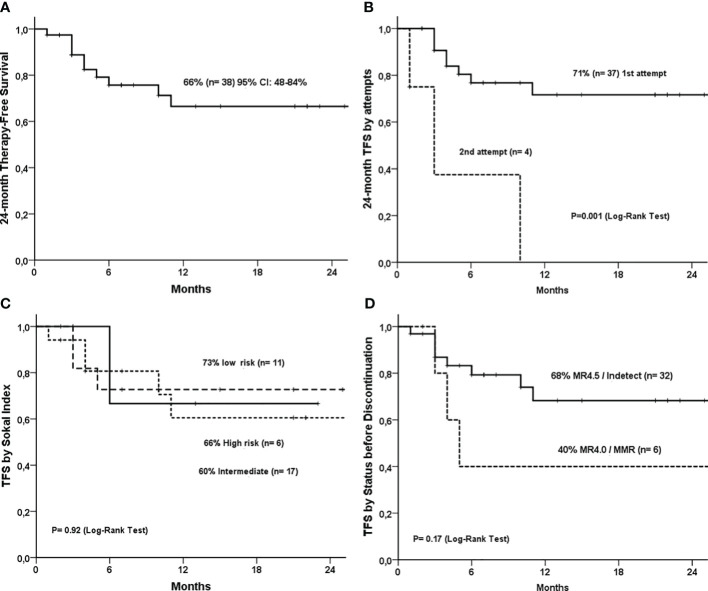
Treatment-free survival curves of 38 CML patients that discontinued TKI. **(A)** 24-month treatment-free survival (TFS); **(B)** 24-month TFS by attempts (first vs second attempt); **(C)** TFS by Sokal Index; **(D)** TFS by molecular response status before discontinuation (MR4.5 vs MR4.0).

Treatment-free survival (TFS) was 66% (95% CI: 48-84%, **Figure 3A**). TFS was 71% in the first-attempt discontinuation group. Three of four cases of second discontinuation attempts relapsed (P<0.0001, **Figure 3B**). There was no difference in TFS, according to the type of *BCR::ABL1* transcripts (**Supplementary Figure 1**), TKI (**Supplementary Figure 2**), and Sokal (**Figure 3C**). There was a trend for a higher TFS in patients with MR4.5 before TKI interruption than in patients with MR4.0 and MMR, but it was not statistically significant (68% versus 40%, P=0.17) (**Figure 3D**). No patient lost hematological response or had disease progression during the study. One patient died from sepsis after a urinary infection (unrelated to CML) during the discontinuation phase.

**Figure 4:**
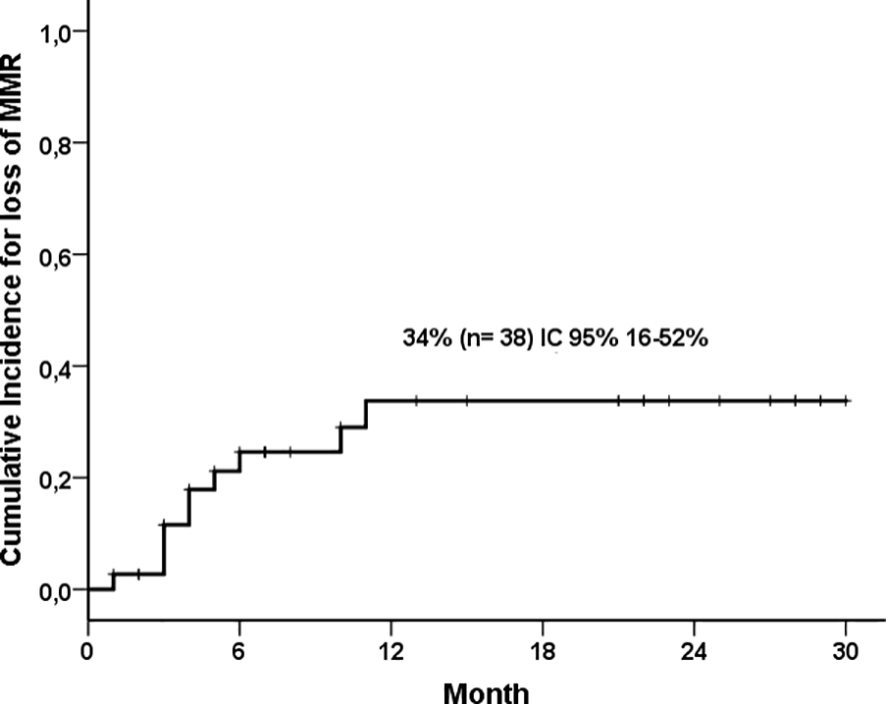
Cumulative incidence of MMR loss.

The cumulative incidence of MMR loss was 34% (95% CI: 16-52%) at 30 months (**Figure 4**). In the multivariate analysis, the independent factors for a longer TFR were the duration of the TKI treatment and the longer duration of MMR before discontinuation (**Table 2**). Among the patients who discontinued therapy, 12 (32%) presented symptoms of withdrawal syndrome in the first four months after discontinuation. Most of the events were of mild intensity (CTCAE Grades 1 and 2), and 8% were grade 3, but they did not require hospitalization or reintroduction of the medication.

**Table 2:** Univariate and multivariate analysis of factors related to molecular relapse.

Variable	HR	95% CI	p-value
Univariate analysis			
Gender= Female	1.03	0.29-3.68	0.95
Sokal (intermediary + high)	1.18	0.29-4.73	0.81
Transcript = b2a2	1.32	0.27-6.41	0.72
MMR duration	0.98	0.97-0.99	0.008
MR4.5 duration	0.98	0.96-0.99	0.01
TKI treatment duration (in months)	0.99	0.98-1.00	0.08
Withdrawal Syndrome	0.60	0.15-2.33	0.46
If MR4.0/MMR on Day 1 of discontinuation	2.81	0.79-9.98	0.11
MR4.5 loss on de-escalation phase	2.28	0.66-7.92	0.19
*BCR::ABL1* ratio fluctuation in the discontinuation phase	4.86	1.03-22.9	0.04
Multivariate analysis			
TKI Treatment Duration (in months)	1.02	1.00-1.04	0.03
MMR duration	0.95	0.92-098	0.004

MMR, major molecular response; MR, molecular response.

## Discussion

The preliminary results of this discontinuation trial, which includes a de-escalation phase, confirmed that a more prolonged TKI treatment and a longer duration of MMR were independent factors for a longer TFR. Fluctuations of MR4.5 during the discontinuation phase were predictive of MMR loss in the univariate analysis, but not confirmed in the multivariate analysis, probably due to the small sample size and short follow-up. The present study is still recruiting patients, which will be accessed in the future.

Treatment-free remission is currently one of the central goals of CML treatment. Several discontinuation trials show it is successful in 50-60% of patients. These studies demonstrated that discontinuation is safe and effective for patients with a deep molecular response, improving patients’ quality of life and reducing treatment costs (11, 12, 16, 28, 29).

Few discontinuation studies included a de-escalation phase before TKI interruption. In most trials, TKI is interrupted abruptly. In our study, TKI was reduced by half of the current dose for six months before discontinuation. Only one case (2.4%) lost MMR during this phase. However, 5/13 patients who lost MR4.5 during de-escalation lost MMR after interruption.

Moreover, the fluctuation of qPCR with loss of MR4.5 in the discontinuation phase was related to a trend of higher rate of molecular relapse in the univariate analysis. In the DESTINY trial, 174 CML patients in stable MMR for the last 12 months went to a phase of 12 months of dose de-escalation to half of the standard TKI dose for 12 months before discontinuation. Seventy-two percent (72%) of the patients who in stable MR4 after the de-escalation phase did not relapse two years after stopping treatment (21). A recent trial randomized 125 CML patients with MR4.5 for over five years in two groups: one for discontinuation and the other for dose de-escalation before TKI discontinuation for 12 months. The molecular recurrence-free survival was lower in the discontinued group compared to the dose de-escalation group (59.9 versus 88.3) p= 0.0002. In the 12 months of dose de-escalation, 8% of patients lost MMR (22).

Regarding other prognostic factors, the present study did not find higher relapse rates in patients with intermediate and high Sokal scores. This finding is in contrast to other studies, such as STIM1, in which the Sokal score was a prognostic factor, leading to a higher relapse rate after discontinuation (13).

Therapy-free survival observed in our study was 66%, similar to other TKI discontinuation trials. TFS was higher in the first attempt group, while most of the patients of the second attempt failed (3 patients). Those patients were retreated with imatinib after the first TFR attempt. The French group was the first to demonstrate the feasibility of a second TKI discontinuation attempt, with a TFR rate of 36% at 24 months (30). In the TRAD study, 59 patients who failed in the first TFR attempt were treated with dasatinib, and 35 achieved MR4.5 or more profound molecular responses and were able to try a second attempt, but 74% lost MMR (31). In the DAstop2 trial, for patients eligible for a second discontinuation attempt, the TFR rate after stopping dasatinib was 46% at 24 months (32).

Eight out of the ten patients lost MMR after discontinuation (80%) within the first six months, and none occurred after 12 months. As observed in most discontinuation studies, relapse tends to occur within the first six months. Twelve (30%) patients have reached the second year of discontinuation and are maintaining MMR. MMR loss is rare after the first year of TFR. In the long-term follow-up of the STIM trial, there were late relapses after two years in 14% of the patients. There was a correlation between fluctuating levels of deep molecular response and late relapse (33). However, due to the short follow-up period in our study, we were unable to analyze this potential correlation.

In the univariate analysis, gender, Sokal score, type of *BCR::ABL1* transcript, and withdrawal syndrome did not impact the TFR rate. The treatment’s longer duration and MMR were independent factors of a lower molecular relapse rate, similar to the findings of the EURO-SKI discontinuation trial (11).

Withdrawal syndrome, which is characterized by musculoskeletal pain after TKI interruption, is expected and occurs in around 30% of patients (34). In our study, we had a similar percentage of patients with withdrawal syndrome. There was no relationship between this adverse event and MMR loss, and it was not necessary to reintroduce TKI in these cases.

In summary, the preliminary results of this study reinforce the importance of the duration of TKI treatment and the longer duration of MMR as prognostic factors for TFR. The fluctuations of molecular responses during the de-escalation and discontinuation phases may require further investigation, particularly with a longer follow-up period, to assess their impact on treatment-free survival more comprehensively.

## Data availability statement

The raw data supporting the findings of this study will be made available by the authors, upon request, and subject to review.

## Ethics statement

The studies involving humans were approved by Comitê de Ética em Pesquisa da Universidade Estadual de Campinas, Campinas-SP, Brazil and Comitê de Ética em Pesquisa em Seres Humanos do Hospital das Clínicas de Ribeirão Preto e da Faculdade de Medicina de Ribeirão Preto (CEP HCRP e FMRP), Ribeirão Preto-SP, Brazil. The studies were conducted in accordance with the local legislation and institutional requirements. The participants provided their written informed consent to participate in this study.

## Author contributions

BM: Data curation, Formal analysis, Investigation, Writing – original draft, Writing – review & editing. GiD: Investigation, Writing – review & editing. LP: Investigation, Writing – review & editing. EM: Investigation, Writing – review & editing, Data curation, Formal analysis. GuD: Investigation, Writing – review & editing. GF: Investigation, Writing – review & editing. IT: Writing – review & editing, Investigation. CD: Writing – review & editing, Investigation. LB: Writing – review & editing, Investigation. VB: Writing – review & editing, Investigation. FC: Writing – review & editing, Investigation. LF: Investigation, Writing – review & editing, Funding acquisition, Resources, Supervision. KP: Investigation, Writing – review & editing, Conceptualization, Data curation, Formal analysis, Funding acquisition, Methodology, Project administration, Resources, Supervision, Writing – original draft.

## Supplementary material

Supplementary Figure 1 Treatment-free survival according to TKI (imatinib vs others) (n=38).

Supplementary Figure 2 Treatment-free survival according to type of BCR::ABL1 transcript (b3a2 vs b2a2) (n=35).
