# Supplementary figures and images for: Kinetics of BCR::ABL1 transcript levels and molecular relapse after tyrosine kinase inhibitors discontinuation in chronic myeloid leukemia patients: preliminary results from the DES-CML study
Source: Front Oncol. 2024 May 8;14:1393191. doi: 10.3389/fonc.2024.1393191 (PMC11109364; doi:10.3389/fonc.2024.1393191)

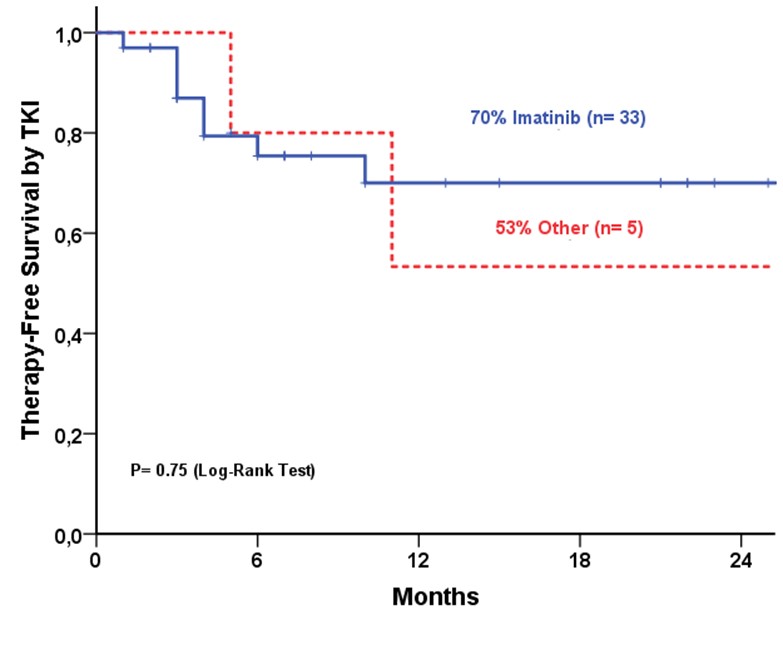

Supplement: Supplementary Figure 1 — Treatment-free survival according to TKI (imatinib vs others) (n=38). [file Image_1.jpeg]

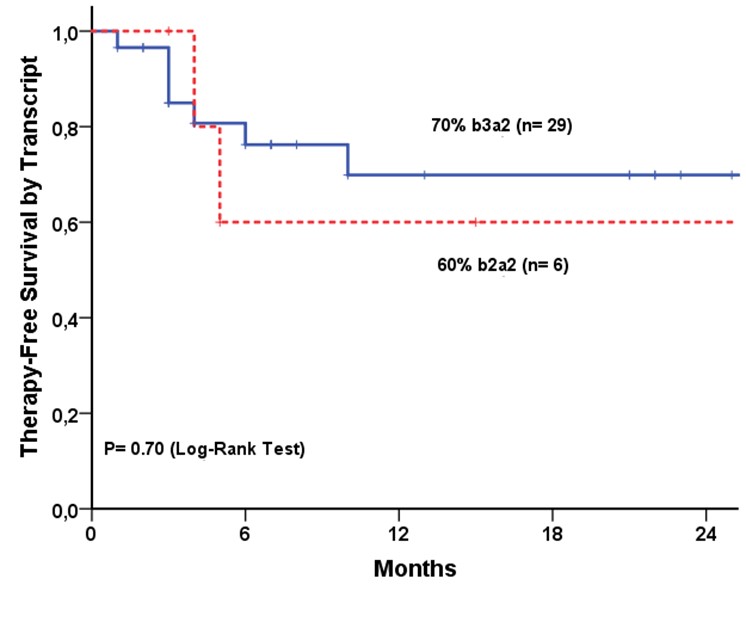

Supplement: Supplementary Figure 2 — Treatment-free survival according to type of BCR::ABL1 transcript (b3a2 vs b2a2) (n=35). [file Image_2.jpeg]
