# Supplementary material for: Kinetics of BCR::ABL1 transcript levels and molecular relapse after tyrosine kinase inhibitors discontinuation in chronic myeloid leukemia patients: preliminary results from the DES-CML study
Source: Front Oncol. 2024 May 8;14:1393191. doi: 10.3389/fonc.2024.1393191 (PMC11109364; doi:10.3389/fonc.2024.1393191)
Supplement: Supplementary file 3 [file Table_1.docx]

**Supplementary Table 1. Dose of TKI at the study entry and dose in the de-escalation phase.**

| **Patient ID** | **TKI** | **TKI dose at study entry (mg)** | **TKI dose in the de-escalation phase (mg)** | **Loss of MMR** |
| --- | --- | --- | --- | --- |
| 1 | Imatinib | 400 | 200 | Yes |
| 2 | Nilotinib | 400 | 200 | Yes |
| 3 | Imatinib | 400 | 200 | Yes |
| 4 | Bosutinib | 300 | 300/200* | No |
| 5 | Dasatinib | 40 | 20 | No |
| 6 | Imatinib | 400 | 200 | No |
| 7 | Imatinib | 300 | 300** | No |
| 8 | Imatinib | 400 | 200 | No |
| 9 | Imatinib | 400 | 200 | Yes |
| 10 | Imatinib | 400 | 200 | Yes |
| 11 | Imatinib | 400 | 200 | Yes |
| 12 | Imatinib | 400 | 200 | No |
| 13 | Imatinib | 400 | 200 | No |
| 14 | Imatinib | 400 | 200 | No |
| 15 | Imatinib | 400 | 200 | No |
| 16 | Dasatinib | 100 | 60 | Yes |
| 17 | Imatinib | 400 | 200 | No |
| 18 | Imatinib | 400 | 200 | No |
| 19 | Imatinib | 400 | 200 | No |
| 20 | Imatinib | 400 | 200 | No |
| 21 | Imatinib | 400 | 200 | Yes |
| 22 | Imatinib | 400 | 200 | No |
| 23 | Imatinib | 400 | 200 | No |
| 24 | Nilotinib | 400 | 200 | Yes |
| 25 | Imatinib | 400 | 200 | Yes |
| 26 | Imatinib | 400 | 200 | No |
| 27 | Imatinib | 400 | 200 | No |
| 28 | Imatinib | 400 | 200 | No |
| 29 | Imatinib | 400 | 200 | Yes |
| 30 | Imatinib | 400 | 200 | No |
| 31 | Imatinib | 400 | 200 | No |
| 32 | Imatinib | 400 | 200 | No |
| 33 | Imatinib | 400 | 200 | No |
| 34 | Imatinib | 400 | 200 | No |
| 35 | Imatinib | 400 | 200 | No |
| 36 | Nilotinib | 800 | 400 | No |
| 37 | Imatinib | 400 | 200 | No |
| 38 | Imatinib | 400 | 200 | No |
| 39 | Imatinib | 400 | 200 | No |
| 40 | Imatinib | 400 | 200 | No |
| 41 | Imatinib | 400 | 200 | No |

*300 mg in alternate days; **300mg alternated with 200mg/day
